# 

**Published:** 1883-01

**Authors:** 


P. W. VAN PEYMA, M. D.

### CONTENTS:

PAGE.

Original Communications:

Calculous Pyelitis. By William Pepper, M. D., LL. D., . . . . . . 241
A Clinical Lecture on Cancer of the Pylorus. By Thomas F. Rochester, M. D. Reported by John H. Pryor, M. D., . 252
Burns and their Treatment. By Chas. A. Wall, M. D., . . . . . . . 257

Clinical Reports:

An Interesting Question in Auscultation. By P. W. Van Peyma, M. D, . . . 270

Editorial Correspondence:

Editorial Correspondence. By A. R. D., . 270

PAGE.

Society Reports:

Buffalo Medical and Surgical Association, . 276
The Buffalo Medical Library Association—Meeting of the Board of Officers and Directors, . . . . . . 281, 282

Editorial:

Law and Malpractice, . . . . . . 282

The Buffalo Medical Library Association, . 284

Editorial Notes, . . . . . . . 285

Reviews: . . . . . . . 287

